# The 3,4-methylenedioxymethamphetamine enhances early visual processing for salient socio-emotional stimuli

**DOI:** 10.1111/ejn.16346

**Published:** 2024-04-18

**Authors:** Connor J. Haggarty, Anya K. Bershad, Mahesh K. Kumar, Royce Lee, Harriet de Wit

**Affiliations:** 1 Department of Psychiatry and Behavioral Neuroscience, University of Chicago, Chicago, Illinois, USA; 2 Pharmacy Practice, Wayne State University, Wayne, Michigan, USA; 3 Semel Institute for Neuroscience and Human Behavior, University of California Los Angeles, Los Angeles, California, USA

**Keywords:** EEG, emotion, faces, MDMA, methamphetamine

## Abstract

The 3,4-methylenedioxymethamphetamine (MDMA) has long been used non-medically, and it is currently under investigation for its potential therapeutic benefits. Both uses may be related to its ability to enhance empathy, sociability, emotional processing and its anxiolytic effects. However, the neural mechanisms underlying these effects, and their specificity to MDMA compared to other stimulants, are not yet fully understood. Here, using electroencephalography (EEG), we investigated the effects of MDMA and a prototypic stimulant, methamphetamine (MA), on early visual processing of socio-emotional stimuli in an oddball emotional faces paradigm. Specifically, we examined whether MDMA or MA enhance the processing of facial expressions, compared to placebo, during the early stages of visual perception. MDMA enhanced an event-related component that is sensitive to detecting faces (N170), specifically for happy and angry expressions compared to neutral faces. MA did not affect this measure, and neither drug altered other components of the response to emotional faces. These findings provide novel insights into the neural mechanisms underlying the effects of MDMA on socio-emotional processing and may have implications for the therapeutic use of MDMA in the treatment of social anxiety and other psychiatric disorders.

## 1 | INTRODUCTION

The 3,4-methylenedioxymethamphetamine (MDMA) is a stimulant-like drug with both nonmedical and potential medical uses. Like other stimulants, it increases dopaminergic and noradrenergic signalling, but relative to other stimulants, it has greater effects on serotonin receptors (Gough et al., 1991; Kalant, 2001; Luethi & Liechti, 2020). MDMA is used recreationally, apparently for its euphoric and empathogenic effects, and it is in Phase 3 trials as an adjunctive treatment, with psychotherapy, for post-traumatic stress disorder (PTSD, Mitchell et al., 2021). Despite the widespread use of the drug and its great promise as a therapeutic agent, the specific behavioural and neural effects of MDMA are not fully understood.

There is considerable evidence that acute doses of MDMA increase sociability, social perception and empathy (Bershad et al., 2016; Heifets et al., 2019; Holze et al., 2020; Kamilar-Britt & Bedi, 2015; Nardou et al., 2019). On self-report measures, the drug increases feelings of empathy, openness and social connectedness and decreases social anxiety and fear (Borissova et al., 2020; Hysek et al., 2014), effects that may enhance social interactions. On behavioural tasks, MDMA reduces sensitivity to negative emotions such as fear or anger (Bedi et al., 2010; Hysek et al., 2014), and reduces social anxiety in some populations (Danforth et al., 2018). It also increases generosity and increases the pleasantness of social touch (Bershad et al., 2019; Kirkpatrick et al., 2015). These laboratory findings complement users’ anecdotal reports that the drug produces prosocial and entactogenic effects (Peroutka et al., 1988).

The neural and hormonal mechanisms by which MDMA acts are not fully understood. Its effects may be related to actions on serotonin or oxytocin systems, both of which are implicated in social behaviour, stress, and social bonding (Bedi et al., 2009; Bershad et al., 2016; Kupers et al., 2018; Thompson et al., 2009). The evidence that its effects are related to oxytocin are mixed, some show that behavioural responses to MDMA are correlated with increases in oxytocin, while others not (Dumont et al., 2009; Francis et al., 2016; Hysek et al., 2014; Kirkpatrick et al., 2014; Vizeli & Liechti, 2017). One recent study (Atila et al., 2023) showed that participants with oxytocin deficiency showed greatly attenuated responses to MDMA, suggesting that adequate baseline oxytocin function may be essential for the drug’s effects.

One way to study the neural effects of MDMA on brain function is to study how the drug alters event-related potentials (ERPs) in response to emotional stimuli. Images of emotional faces in an oddball task elicit three distinctive ERP components: N170, P300 and mismatch negativity (MMN). The N170 component is a negative waveform that is thought to reflect the processing of facial features and the structural encoding of faces (Bentin et al., 1996). The P300 component is a larger positive waveform that is thought to reflect attention allocation and cognitive processing (Carretié et al., 1997). Finally, the MMN component is a negative waveform that occurs approximately 200–300 ms after stimulus onset and appears to respond to novel stimuli (Stefanics et al., 2014). The effects of a drug on these components in response to emotional stimuli may advance our understanding of how the drug alters responses to emotionally salient stimuli.

Here we conducted a double-blind study examining effects of MDMA and MA, compared to placebo, on these EEG responses to emotional faces. We contrasted MDMA to a prototypic amphetamine, MA, which is thought to lack the strong prosocial effects of MDMA (Bershad et al., 2016). Healthy young adults received MDMA (100 mg), MA (20 mg) or placebo during three sessions. We measured electrophysiological responses to emotional and neutral faces using an emotional oddball task. We hypothesized that MDMA, but not MA, would enhance the N170 and P300 ERP components associated with socio-emotional processing specifically for more salient stimuli. The aim of this study was to determine how MDMA affects the brain’s processing of social stimuli, including positive, negative and neutral faces. We also sought to determine whether the effect of MDMA differs from the effect of a prototypic stimulant, methamphetamine.

## 2 | MATERIALS AND METHODS

### 2.1 | Design

The study used a double-blind, within-participant design in which healthy adults received MDMA (100 mg), MA (20 mg) and placebo (PLC) on three separate sessions. At the expected time of peak drug effect participants engaged in an oddball task during which we recorded EEG responses to positive, negative and neutral faces. ERP components related to different aspects of emotional and cognitive processing were recorded. Participants also completed self-report measures of the drugs’ effects. The study was approved by the University of Chicago internal review board and all procedures were in line with the declaration of Helsinki.

### 2.2 | Participants

Healthy men and women (*N* = 25) aged 18–35 were recruited from the university and surrounding neighbourhoods. Eligibility was determined first by online screening and then by clinical interview and physical examination. Inclusion criteria consisted of a minimum high school education, fluency in English, BMI 18–26, and good health. Exclusion criteria included use of prescription medications, history of cardiac disease or high blood pressure and previous negative experience with MDMA or hallucinogenic substances. Participants had to report between 4 and 40 previous experiences of MDMA to qualify for the study. Women who were not on oral contraceptives were tested only during the follicular phase (1–12 days from menstruation; White et al., 2002).

### 2.3 | Procedure

Participants first attended an orientation to understand the procedures, provide informed consent and practice the tasks from the experimental sessions. They were instructed to abstain from drugs and alcohol for 24 h before their sessions. They were told they would receive a *sedative (*e.g.*, Valium)*, *stimulant (*e.g.*, MDMA or amphetamine)* or *placebo* drug in each session. The study was approved by the local institutional review board.

The three, 4-h drug sessions were conducted from 9 AM to 1 PM, separated by at least 4 days (*M* = 7.5 days). Upon arrival at the laboratory, participants completed a breathalyser and urine sample to test for recent drug use (CLIA waived Instant Drug Test Cup, San Diego, CA; amphetamine, cocaine, oxycodone, THC, PCP, MDMA, opiates, benzodiazepines, barbiturates, methadone, methamphetamine and buprenorphine), alcohol use (Alcosensor III, Intoximeters, St. Louis, MO) and pregnancy (in females; Aimstrip, Craig Medical, Vista, CA). They completed pre-capsule questionnaires and cardiovascular measures, measures that were repeated 60, 90, 180 and 240 min after the capsule. They ingested capsules containing dextrose (placebo), MA (20 mg) or MDMA (100 mg) under double-blind conditions. Thirty minutes after taking the capsule participants’ EEG electrodes were placed, and recording began about 60 min after the capsule. Resting state EEG was determined first, and this was followed by three tasks completed in randomized order. Here we report data on an emotional oddball task assessing responses to happy, angry, and neutral faces (Raz et al., 2014). The EEG measures were obtained from 60 to 150 min post-capsule. After the EEG electrodes were removed, participants rated the arousal and valence of the faces they viewed during the EEG task. Participants left the laboratory after the final measure, 240 min post capsule.

#### 2.3.1 | Drugs

MDMA in powdered form (100 mg; Organix Inc, MA) was placed in opaque size 00 capsules with lactose filler. MA tablets (5 mg, total dose 20 mg; Desoxyn, Lundbeck) were placed in an opaque size 00 capsule with dextrose filler, and placebo capsules contained only dextrose.

### 2.4 | Self-report measures

1. The Drug Effects Questionnaire (DEQ; Morean et al., 2013) The DEQ consists of 100-point visual analog scales (VAS) describing responses to the drug. Here we focused on the questions ‘Do you FEEL any drug effects right now’ rated from ‘*Not at all*’ (0) to ‘*Very Strong Effect*’(100), ‘Do you LIKE the effects that you are feeling now?’ (‘*Not at all*’ [0] to ‘*Very much*’ [100]) and ‘Would you like more of what you consumed, right now?’ (‘*Not at all*’ [0] to ‘*Very much*’ [100]).
2. Visual Analogue Scales (VAS). The VAS consisted of 14 words describing drug-like experiences. Here we present data from the words ‘Sociable’ and ‘Friendly’. Participants rated on a 100-point scale how strongly they felt those feelings (‘*not at all*’ to ‘*extremely*’) at each of the five time points during each session.
3. The Session End Questionnaire (SEQ). The SEQ consists of questions relating to the drug received during the session. Participants indicated how pleasant they found the experience (from ‘*dislike*’ [0] to ‘*like very much*’ [100]) and what they thought they had received (i.e., Valium, Ketamine, Amphetamine, MDMA, LSD, Placebo).

### 2.5 | EEG measures

#### 2.5.1 | Data collection and processing

A 64-channel electro-geodesic net was used (Magstim, EGI). Electrodes were soaked in a saline solution and then placed on the head using measurements from nasion to inion and mastoid to mastoid. EEG data was acquired continuously, amplified and digitized using Netstation software, and sampled online at 1024 Hz with impedances below 50kΩ. Offline EEG recordings were analysed using EEGLab (Delorme & Makeig, 2004) was first down-sampled to 512 Hz then high pass filtered (1 Hz), and low pass filtered (60 Hz, −12 dB/octave) to remove extraneous high and low-frequency noise. Data were visually inspected for movement and electronic artefact, that is, periods of data with excessive noise affecting all electrodes. PICARD Independent Components Analysis (Frank et al., 2022) were performed to correct for EEG artefacts including blinks, horizontal and vertical eye movements, muscle movement and EKG signal only. Data were segmented from −200 to 1000 ms and baseline corrected following stimulus presentation for each of the stimulus types. Before the values were averaged, artefact detection was used to classify any segment of data with ±100 μV remaining and segment breaks from earlier cleaning. Further, a threshold of >80% was selected for inclusion in averaging for ERPs, all participants met this threshold.

##### Emotional oddball task

To assess the neural responses to emotional stimuli, participants completed an emotional oddball task (Raz et al., 2014; Schlüter & Bermeitinger, 2017). The task consisted of 300 stimulus presentations, of which 80% were cartoon faces, and 20% were human faces. The stimuli were presented in three blocks of 100 stimuli consisting of the frequent cartoon image interspersed with infrequent angry, happy, or neutral faces. Emotional faces were not mixed within blocks to allow for individual consideration of each valence. Subjects were instructed to respond on the left key on a button box when they saw a human face and, on the right when they saw a cartoon face. ERPs were recorded during each stimulus presentation.

### 2.6 | Behavioural measure

#### 2.6.1 | Face ratings task

Following the EEG session participants completed a ratings task in which they rated the 6 faces presented in the oddball task for valence and arousal. Valence and arousal were rated on a Likert scale from −4 (*Very Negative/Not at all*) to +4 (*Very Positive/Extremely*). Participants were told to indicate how positive or negative they perceived each face (valence) and how strongly they felt that emotion (arousal) (Figure S1).

### 2.7 | Analysis

1. Subjective and behavioural measures were assessed with analysis of variance using peak change score from baseline, using individual paired samples *T*-tests to measure the differences between Placebo versus MA and Placebo versus MDMA. Peak drug effects were calculated by subtracting the baseline values from the highest or lowest value during the session.
2. Faces Ratings Task (supplementary results). Participants rated the valence and arousal of each of the six faces seen in the emotional oddball task (male and female; angry, happy, neutral). These ratings were analysed using two 3 × 2 (emotion × MDMA [vs. PLC]; emotion × MA [vs. PLC]) ANOVAs.
3. Emotional Faces Oddball Task. The N170 was measured at electrode PO8, extracting the mean peak from 150 to 200 ms post stimulus onset (Bentin et al., 1996). The P300 was measured at electrode parietal (Pz) extracting the mean peak between 300 and 400 ms (Carretié et al., 1997). The MMN was measured using electrodes Fz between 240 and 350 ms (Stefanics et al., 2014). While electrodes of interest were selected based on these previous studies, specific time windows were chosen through visual inspection of grand average ERPs. For each of these peaks of interest, two 3 (emotion) × 2 (Drug condition; Drug vs. PLC) repeated measures ANOVAs were conducted. We also examined ERP responses to the frequent cartoon faces for MMN to compare frequent versus infrequent stimuli. Initial analyses (supplementary materials) revealed that responses to the (infrequent) human faces differed from markedly from responses to the (frequent) cartoon face on all three ERP measures. The final analyses were conducted only with the human faces, comparing MDMA and MA versus PLC separately.

## 3 | RESULTS

### 3.1 | Demographics

Participants were 17 men and eight women, mean age of 27.4 years, most of whom had completed partial college (Table 1). Participants had previously taken MDMA a mean of 7.1 times. None of the subjects were cigarette smokers. Sixty-four percent of participants correctly identified Placebo, 20% MA and 52% MDMA, showing the effectiveness of the blinding (Table S1).

### 3.2 | Subjective measures

**FIGURE 1:**
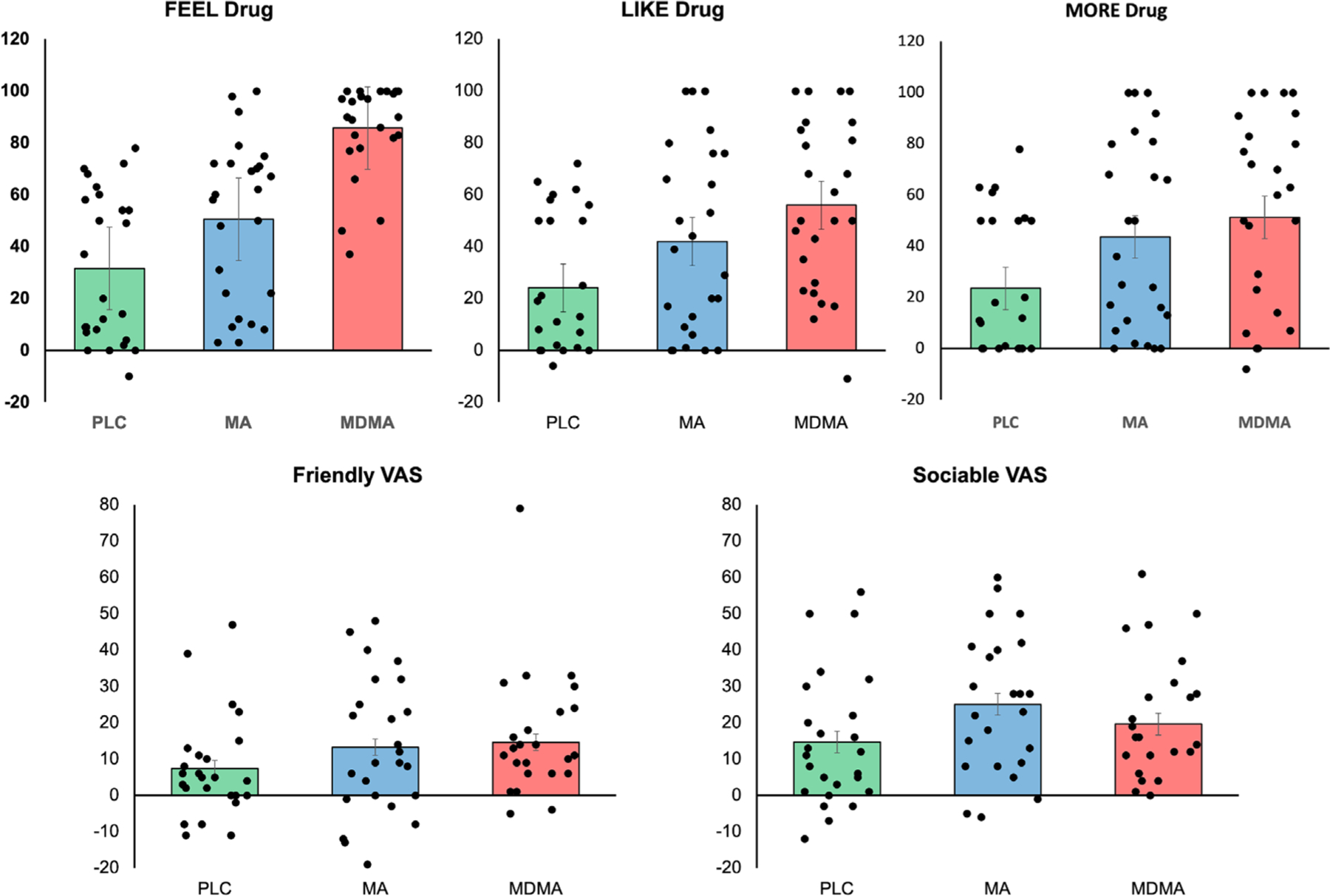
Mean and standard error of the mean (SEM) for ratings of feel drug, liking, wanting more, visual analog scales (VAS) ratings of friendly and sociable for placebo (PLC) (green), methamphetamine (MA) (blue) and 3,4-methylenedioxymethamphetamine (MDMA) (red) sessions. MDMA significantly increased ratings for feeling the drug, liking drug effects, wanting more of the drug, and how friendly participants felt, compared to placebo, whereas MA significantly increased ratings of feeling the drug, wanting more drug, feeling friendly and sociable.

Both MDMA and MA increased ratings of Feel Drug compared to PLC (MDMA, *t*(24) = 7.83, *p <* .001; MA, *t*(24) = −2.03, *p* = .05). MDMA increased ratings of drug liking relative to PLC (*t*(24) = −3.93, *p* = .001) but MA did not differ from PLC (t(24) = −2.00, *p* = .06). Both MDMA and MA increased wanting more drug ratings compared to PLC (MDMA, *t*(24) = −3.32, *p* = .003; MA, *t*(24) = −2.38, *p* = .03). For VAS ratings, MDMA significantly increased feelings of friendliness compared to PLC (*t*[23] = −2.06, *p* = .05), as did MA (*t*[23] = −2.15, *p* = .04). In contrast, MA but not MDMA increased feelings of sociability (*t*(24) = −2.14, *p* = *04*) (Figure 1).

### 3.3 | Session end questionnaire

On the end of session questionnaire, 64% of participants correctly identified PLC, 20% of participants correctly identified MA as a stimulant, and 52% correctly identified MDMA (full breakdown in supplementary results). Subjects reported liking MDMA (mean = 78.0) and MA (mean = 71.2) more than PLC (mean = 48.2).

#### 3.3.1 | Drug effects on ERP peaks

**FIGURE 2:**
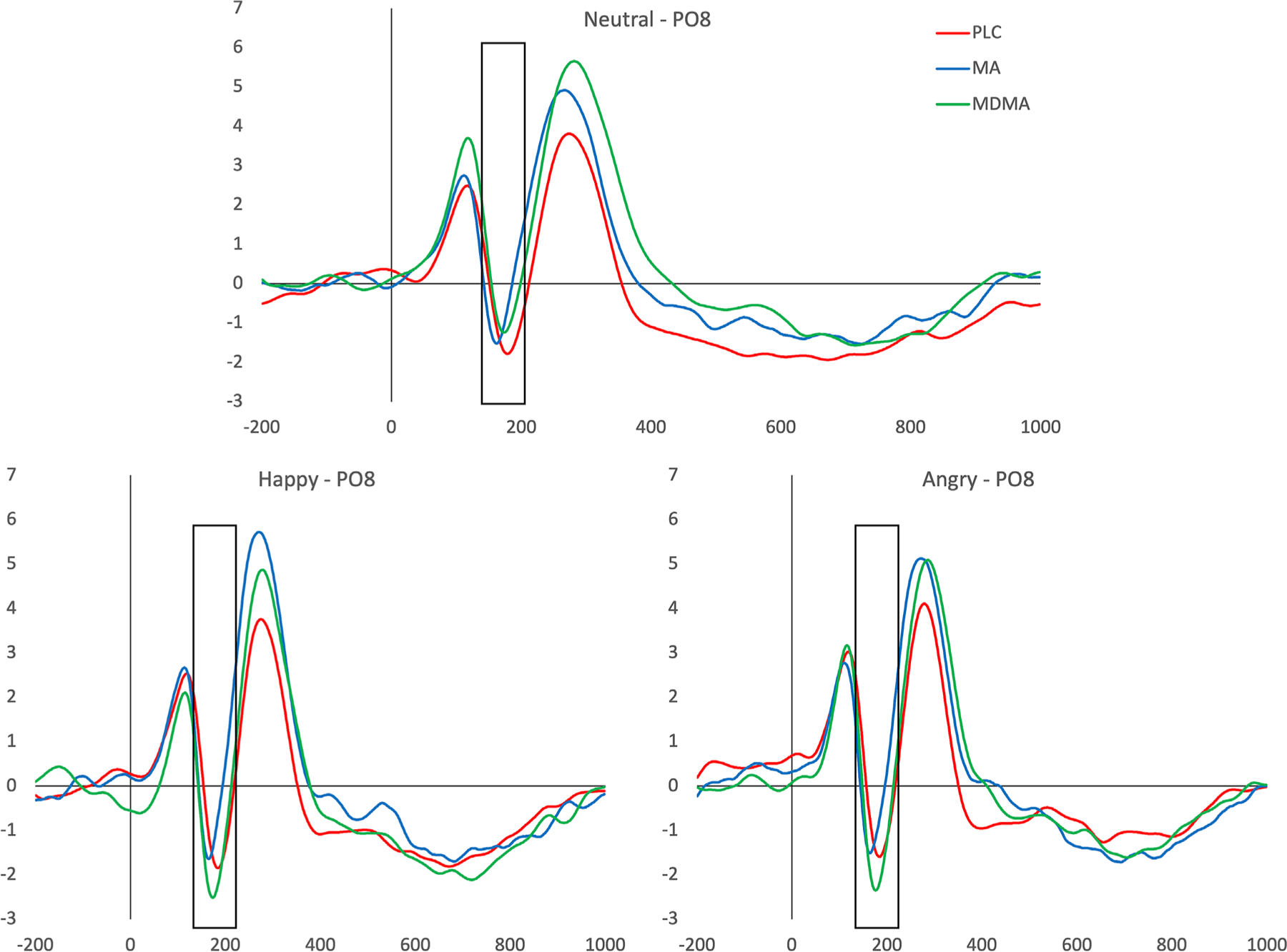
*N170* event related potentials (ERPs) at electrode PO8 (shaded area) for happy, angry and neutral faces. ERPs are shown for placebo (PLC) (blue), methamphetamine (MA) (red) and 3,4-methylenedioxymethamphetamine (MDMA) (green) faces. MDMA significantly increased N170 peak amplitude in response to happy and angry faces, but not neutral faces when compared with PLC.

N170—Face processing (Figure 2). MDMA significantly increased N170 peak amplitude compared to PLC specifically for happy (MDMA/PLC; *M* = −2.27 μV/M = −1.04 μV) and angry (MDMA/PLC; *M* = −2.09 μV/M = −1.31 μV) faces (significant drug × emotion interaction, *F*[2,86] = 4.49, *p* = *.01*, η_*p*_^2^ = .1). Regardless of whether participants received MDMA or PLC, the N170 amplitude was also greater with happy and angry faces compared to neutral faces (main effect of emotion *F* [2,88] = 7.86, *p* = *.001*, η_*p*_^2^ = .15). MA did not affect the N170 peak amplitude, compared to PLC (no significant interaction between drug and emotion, *F*[2,86] = .31, *p > .05*, η_*p*_^2^ = .007). In this MA versus PLC analysis, emotion did not affect peak N170 amplitude here (no main effect of emotion, *F*[2,86] = 1.21, *p > .05*, η_*p*_^2^ = .03).

**FIGURE 3:**
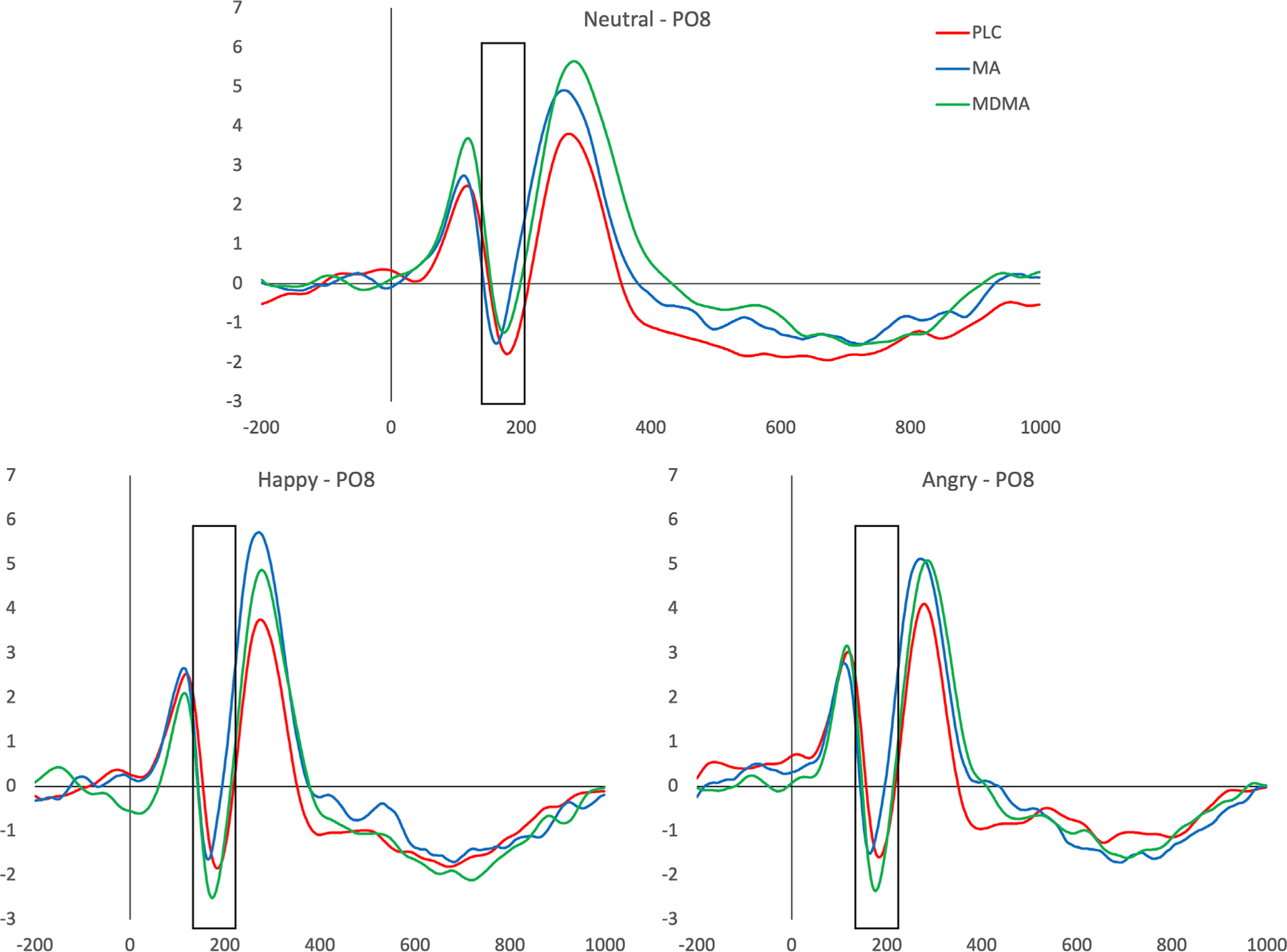
Event related potentials (ERPs) at electrode Pz. Shaded area represents P300 peak amplitudes for happy (top), angry (bottom left) and neutral (bottom right) faces with ERPs shown for placebo (PLC) (blue), methamphetamine (MA) (red) and 3,4-methylenedioxymethamphetamine (MDMA) (green) sessions. Neither MDMA nor MA affected the peak amplitude of P300 ERPs. There was also no effect of emotion on the P300 response.

P300—Emotion processing (Figure 3). MDMA did not affect the P300 peak amplitude compared to PLC (no significant interaction between drug and emotion [*F*{2,86} = 1.36, *p > .05*, η_*p*_^2^ = .05] or main effect of drug [*F*{2,86} = 3.37, *p* = *.07*, η_*p*_^2^ = .07]). Similarly MA did not affect P300 (no significant interaction with emotion [*F*{2,88} = 2.20, *p > .05*, η_*p*_^2^ = .05] or main effect of drug [F{2,86} = .14, *p > .05*, η_*p*_^2^ = .003]). Emotion was not significantly related to P300 peak for either MDMA or MA versus PLC (MDMA: *F*[2,86] = 2.02, *p > .05*, η_*p*_^2^ = .05; MA: F[2,86] = .72, *p > .05*, η_p_^2^ = .02) (Figure 3).

**FIGURE 4:**
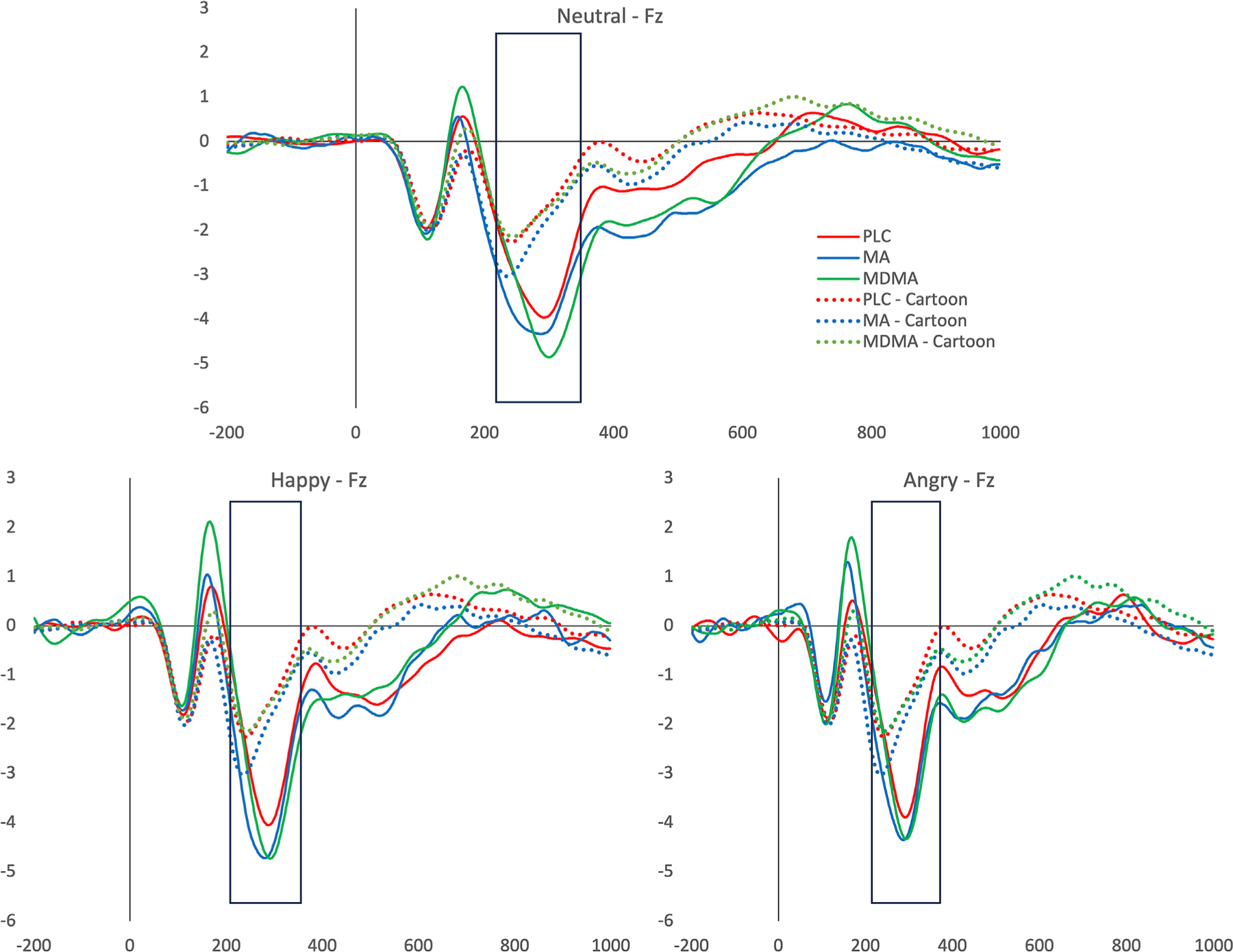
Event related potentials (ERPs) at electrode Fz. Shaded area represents mismatch negativity (MMN) peak amplitudes for happy (top), angry (bottom left), neutral (bottom right) and cartoon (dashed) faces with ERPs shown for placebo (PLC) (blue), methamphetamine (MA) (red), 3,4-methylenedioxymethamphetamine (MDMA) (green) sessions. MDMA and MA did not affect the peak amplitude of MMN however MMN was present for human faces compared to cartoon faces. Furthermore, MMN was not affected by the emotion of the face shown.

MMN—Novelty processing (Figure 4). MDMA did not affect MMN amplitude compared to PLC (no interaction between drug and emotion [*F*{3129} = .91, *p > .05*, η_*p*_^2^ = .02] and no main effect of drug [*F*{1,43} = .48, *p > .05*, η_*p*_^2^ = .01]). MA also did not affect the MMN peak amplitude (no significant interaction [*F*{3129} = .51, *p > .05*, η_*p*_^2^ = .01] and no main effect of drug [*F*{1,43} = .90, *p > .05*, η_*p*_^2^ = .02]). The type of face shown, however, did affect peak amplitude in PLC and MDMA sessions (*F*[3129] = 39.00, *p < .001*, η_*p*_^2^ = .48) with Human faces resulting in a significantly greater MMN than Cartoon faces (all ps < .001). This was expected because MMN signals novel/infrequent stimuli. Similarly, in the analysis of MA versus PLC face type significantly affected MMN amplitude whereas drug type did not (*F*[3129] = 37.14, *p < .001*, η_*p*_^2^ = .46) again with human faces resulting in a significantly greater MMN than the cartoon face (all ps < .001) (Figure 4).

## 4 | DISCUSSION

The current study investigated the effects of two drugs, MDMA and MA, compared to placebo, on evoked potential responses to emotional stimuli in healthy adults. The two drugs produced their expected subjective effects. MDMA increased the N170 peak amplitude for happy and angry faces compared to neutral faces, but MA did not have this effect. The N170 is thought to reflect processing of facial features and is sensitive especially to emotional faces. Neither drug significantly altered P300 or MMN evoked potentials which are thought to reflect attention allocation and cognitive processing (P300) and responses to novelty (MMN).

Both MDMA and MA had expected effects on subjective and behavioural measures. Both MDMA and MA increased ratings of feeling and liking the drug effect and wanting more of the drug. Both drugs also increased feelings of friendliness whereas only MA increased feelings of sociability. The lack of effect of MDMA on sociability was surprising considering some previous reports (Kirkpatrick et al., 2014; Wardle, Kirkpatrick & de Wit, 2014), and because MDMA is often described as a prosocial drug. It is known that social context can influence responses to MDMA (Kirkpatrick & de Wit, 2015), so it is possible that the solitary laboratory environment in the present study prevented the drug from producing feelings of sociability. As expected, participants rated happy faces more positively than neutral faces, and angry faces more negatively than neutral faces. However, neither MDMA nor MA altered these ratings.

The main finding in this study was that MDMA, but not MA, enhanced the N170 amplitude in response to salient emotional faces. The drug increased N170 signals with happy and angry, but not neutral, faces. To our knowledge, the effects of MDMA and MA on N170 ERPs have not previously been examined. The N170 is thought to represent early structural encoding of face stimuli, especially faces expressing emotions (Bentin et al., 1996). Emotional states can enhance the peak of this ERP (Blau et al., 2007; Qiu et al., 2017). Interestingly, the prosocial hormone oxytocin reportedly increases N170 responses to faces, especially sad faces (Peltola et al., 2018). The finding that the pro-social drug MDMA, like oxytocin, increased N170 responses to emotional faces is consistent with the idea that the N170 signals early social approach/avoidance behaviours (Schindler & Bublatzky, 2020). This effect on N170 suggests that MDMA affects early visual processing of socially salient stimuli, before it produces other cognitive or emotional processes (Bershad et al., 2016; Hysek et al., 2014; Kirkpatrick et al., 2014; Wardle & de Wit, 2014). MDMA has also been shown to increase the recall of positive and emotional memories but to not affect neutral ones, which is consistent with the idea that MDMA indeed enhances emotional processing (Doss et al., 2018). MA, which has less pronounced effects on social processes (Bershad et al., 2016), did not affect the N170. It is important to consider also that there is evidence to suggest that long term MDMA use may impact social functioning differently. For example, Wunderli et al. (2018) reported that long term MDMA users showed enhanced cognitive empathy but not emotional empathy. Comparatively Carlyle and colleagues (2019) showed that both cognitive and emotional empathy were enhanced in the MDMA users.

Contrary to our expectations, neither MA nor MDMA affected the P300 peak amplitude. The P300 is thought to reflect attention allocation and cognitive processing and thus might be expected to be greater with more salient stimuli (emotional faces), and enhanced by drugs that increase attention. In the present study, the P300 was not affected by either emotions or either of the two drugs, although the P300 was smaller with cartoon faces compared to human faces. Although the cartoon faces were much more frequent than the human faces, it may also be that the P300 is sensitive to faces but not emotions. The P300 is thought to reflect cognitive processing related to attention and orientation (Morgan et al., 2008; Mueller et al., 2017). The lack of effect of either MDMA or MA on P300 is somewhat surprising, considering that both drugs are considered stimulants, which are thought to improve attention. Moreover, MDMA has been shown to increase visual attention to happy faces compared to other emotions (Bershad et al., 2019), but this effect was not detected here with related EEG measures. Thus, the effects of MDMA and MA attention likely vary, depending on the measures that are used to assess attention, as well as the doses and participants studied (Cami et al., 2000; Silber et al., 2012).

The MMN ERP was not affected by either MDMA or MA. Because the MMN is thought to signal a response to novel stimuli, this suggests that neither drug increased neural response to novelty. Regardless of the drug, the MMN ERP was greater with infrequent human faces than with frequent cartoon faces, supporting the idea that the MMN is an indicator of novelty. However, neither drug appeared to affect the neural indicator of novelty of the emotional faces. Although there is indirect evidence that stimulants increase the salience of stimuli (Chaudhri et al., 2007; Taylor & Robbins, 1984), few other studies have examined the effects of stimulants specifically on MMN amplitude. In the present study, neither MDMA nor MA altered the brain’s response to novelty in the context of emotional stimuli, even though MDMA altered stimulus salience as measured by the N170. Few studies have been conducted on the effects of other drugs on MNN. In one of few such studies, single doses of the selective serotonin uptake inhibitor escitalopram significantly increased MMN, without affecting P300 amplitude (Wienberg et al., 2010). Whether these ERP-related differences are related to the drugs’ behavioural effects remains to be determined. Further research is needed to determine whether, or how, stimulant drugs affect this neural indicator of novelty.

The study had several limitations. The sample was homogeneous, limited to healthy men and women aged 18–35, who had previously used MDMA. Thus, the results may not be generalizable to a more heterogeneous population including those with greater or lesser drug use histories. While the sample size was within the typical size for studies involving EEG recordings, more subtle effects would likely be detected with a larger sample. The study used only one dose of MDMA and MA, making it difficult to compare across drugs. That is, higher or lower doses of either drug may produce different effects, and future studies with dose responses are critical. MA is seven times more potent than MDMA at inhibiting noradrenaline transporters (Simmler et al., 2013); therefore, it is important to consider that a higher dose of MA would be required to appropriately compare these drugs together; for this reason, the drugs were compared separately compared to PLC. In future studies, it would be beneficial to use more than one dose or match doses for their potency on one variable of interest. Another limitation is the 4-day washout period between sessions. Although this interval resulted in undetectable levels of MDMA on urine drug tests, it is shorter than the five half-lives typically used in pharmaceutical trials. Moreover, it is not clear how long an interval is needed for serotonin levels to return to normal after acute administration of the drug. Therefore, in future studies, it will be important to extend the inter-session interval. Finally, the study was limited by the task that was used, and it is possible that the emotional faces oddball task is not optimal for detecting the effects of these drugs. In future studies, more complex studies of social behaviour and motivation may parse the effects that MDMA is having on these processes.

The present findings have implications for MDMA-assisted therapy. The increased neural response to the sensory component of viewing faces could contribute to the therapeutic alliance between patients and their therapists. By increasing attention to facial emotional cues, the drug may increase interpersonal connection in the therapeutic environment. MDMA may also facilitate the identification and processing of emotions, allowing patients to build trust, and engage more deeply with their emotional experiences. Further studies of this kind are needed to understand the brain mechanisms underlying the behavioural effects of MDMA and other pro-social drugs.

The main finding in this study was that MDMA, but not MA, increased the N170 peak amplitude for angry and human faces, compared to neutral faces. This finding is consistent with evidence that the N170 is selective for faces and sensitive to emotion. The effect of MDMA on N170 is consistent with both the known function of this ERP, with evidence that MDMA affects responses to social stimuli. An interesting and important future direction is to clarify whether MDMA equally affects both positive and negative emotional stimuli, as the present data suggest. This would have implications for its use in therapeutic settings. The finding that MA did not have similar effects suggests MDMA differs from other stimulants in the processing of social stimuli, although this conclusion must await testing with a full range of doses.

## Supplementary Material

Supp info

## Figures and Tables

**TABLE 1:** Demographic information and nonmedical drug use (*N* = 25).

Sex (number M/F)		(17, 8)
Age (mean and SEM)		27.4 (4.2)
BMI (mean and SEM)		23.1 (2.8)
Years in education (mean and SEM)		16.1 (1.7)
Ethnicity (percent of sample)	Caucasian/White	58%
	African American	13%
	Asian	17%
	Other/more than one	13%
Recent drug use (last 30 days)	Alcohol (drinks/week)	6.3 (5.0) [*N* = 23]
	Alcohol (drinks/occasion)	2.7 (1.5) [*N* = 23]
	Alcohol (occasions/week)	2.0 (1.6) [*N* = 23]
	Caffeine (cups/day)	1.4 (1.0) [*N* = 20]
	Cannabis (uses/30 days)	6.5 (7.9) [*N* = 19]
Lifetime MDMA (uses)		7.1(10.1) [*N* = 25]

*Note*: Drug use data are represented as mean (SEM) of uses only for the participants who reported ever using the drug [*N*].

## Data Availability

Data and figures can be freely accessed on FigShare 10.6084/m9.figshare.25393192.

## Abbreviations:

EEG: electroencephalography
ERP: event related potential
MA: methamphetamine
MDMA: 3–4-dioxymethamphetamine
MMN: mismatch negativity
PLC: placebo
