# Supplementary material for: The 3,4-methylenedioxymethamphetamine enhances early visual processing for salient socio-emotional stimuli
Source: Eur J Neurosci. Author manuscript; Available in PMC 2024 Sep 17. (PMC11406194; doi:10.1111/ejn.16346)
Supplement: Supp info [file NIHMS2015839-supplement-Supp_info.docx]

**Supplementary analyses.**

**Supplementary Table 1.** Session end questionnaire percentage drug identifications, showing what participants thought they had received on each experimental session.

|  |  | **Drug Identification** | | | | | |
| --- | --- | --- | --- | --- | --- | --- | --- |
|  |  | **Valium** | **Ketamine** | **Amphetamine** | **MDMA** | **LSD** | **Placebo** |
| **Session** | **Placebo** | 28 | 4 | 4 | 0 | 0 | 64 |
|  | **MA** | 12 | 4 | 20 | 44 | 0 | 20 |
|  | **MDMA** | 4 | 16 | 20 | 52 | 8 | 0 |

**Faces Ratings.**

Neither MDMA nor MA significantly altered valence ratings of the human faces compared to PLC (MDMA F(1,42)= 1.18, *p>.05,* η_p_^2^=0.03; MA F(1,42)= 0.61, *p>.05,* η_p_^2^=0.002). However, participants rated Happy faces more positively than PLC and Angry faces more negatively than placebo regardless of drug (main effect of Emotion in MDMA comparison, F(2,84)= 516.43, *p<.001,* η_p_^2^=0.93; main effect of Emotion in MA comparison, F(2,84)= 460.59, *p<.001,* η_p_^2^=0.92) (Supplementary Figure 1).

Similarly, neither drug significantly altered ratings of Arousal for human faces compared to PLC (MDMA, F(1,41)= 0.85, *p>.05,* η_p_^2^=0.02; MA, F(1,42)= 0.10, *p>.05,* η_p_^2^=0.002), but ratings were significantly related to Emotion in both comparisons (main effect of emotion, MDMA vs PLC, F(2,84)= 14.69, *p<.001,* η_p_^2^=0.26, MA vs PLC, F(2,84)= 22.79, *p<.001,* η_p_^2^=0.35).


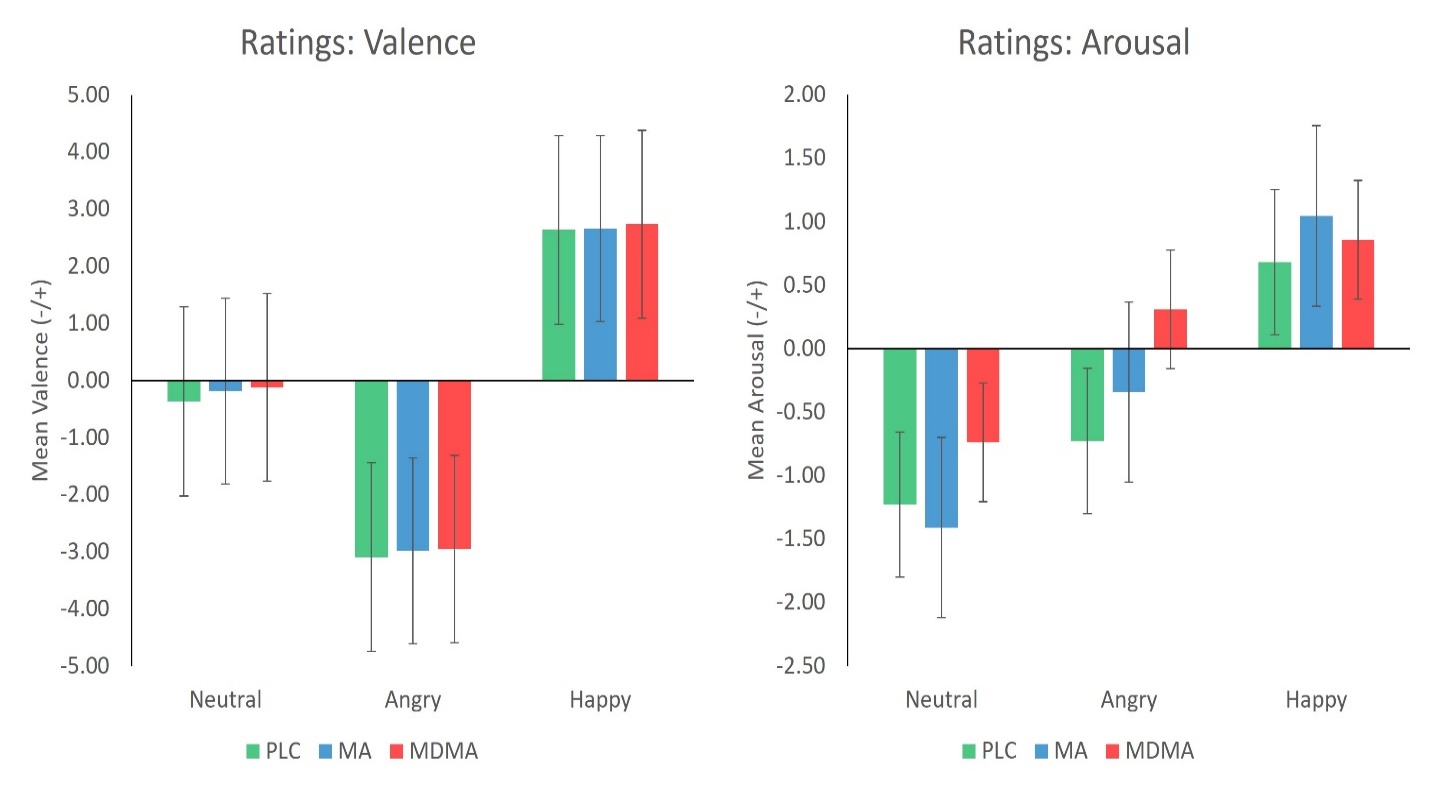


**Supplementary Figure 1.** Mean (sem) ratings of Valence and Arousal with Neutral, Angry and Happy faces after PLC, MDMA and MA. Neither drug significantly altered Valence or Arousal ratings compared to placebo. Angry faces were rated more negatively than Neutral faces, Happy faces more positive than Neutral faces, and Neutral faces were rated as the least arousing.

**Supplementary EEG analyses.**

*Human vs Cartoon Faces.* Neither drug affected N170 amplitude when both human and Cartoon faces were included in the analysis (no main effect of Drug, F(2,65)= 0.79, *p>.05,* η_p_^2^=0.007). However, human faces regardless of the emotion, resulted in a significantly greater N170 than Cartoon faces (main effect of Face, F(3,195)= 28.46, *p<.001,* η_p_^2^=0.30). Within the human faces, Happy (M=-1.57), Angry (M=-1.68) and Neutral faces (M=-0.96) elicited significantly greater N170 peaks than Cartoon faces (M=0.14). Similarly, neither Drug influenced P300 peak amplitude (no main effect of Drug, F(2,65)= 1.80, *p>.05,* η_p_^2^=0.05), but Cartoon faces had a significantly smaller P300 than Human faces (main effect of Emotion, F(3,195)= 54.88, *p<.001,* η_p_^2^=0.46).
